# The Hazardous Level of Heavy Metals in Different Medicinal Plants and Their Decoctions in Water: A Public Health Problem in Brazil

**DOI:** 10.1155/2020/1465051

**Published:** 2020-03-12

**Authors:** Paula F. S. Tschinkel, Elaine S. P. Melo, Hugo S. Pereira, Kassia R. N. Silva, Daniela G. Arakaki, Nayara V. Lima, Melina R. Fernandes, Luana C. S. Leite, Eliane S. P. Melo, Petr Melnikov, Paulo R. Espindola, Igor D. de Souza, Valdir A. Nascimento, Jorge L. R. Júnior, Ana C. R. Geronimo, Francisco J. M. dos Reis, Valter A. Nascimento

**Affiliations:** ^1^Group of Spectroscopy and Bioinformatics Applied to Biodiversity and Health, School of Medicine, Postgraduation Program in Health and Development in the Midwest Region, Faculty of Medicine, Federal University of Mato Grosso do Sul, Campo Grande, Mato Grosso do Sul, Brazil; ^2^Institute of Chemistry of the Federal University of Mato Grosso do Sul, Campo Grande, Brazil

## Abstract

The determination of Cd, Co, Cr, Cu, Fe, Na, Zn, and Pb by inductively coupled plasma-optical emission spectrometry (ICP OES) was performed on dry matter and decoctions of the medicinal plants *Cordia salicifolia*, *Chiococca alba* (L.) Hitchc., and *Echites peltata* used as an appetite suppressant and diuretic in Brazil. The accuracy of the measurements was analyzed by the spike recovery test. Results showed that the concentration of these seven metals (Cd, Co, Cr, Cu, Fe, Na, and Zn) in dry plant samples is below the oral concentration of elemental impurities established by the United States Pharmacopoeia Convention (USP). However, there are no concentration limits for Fe, Na, and Zn established by the USP in drug substances and excipients. Levels higher than the recommended value by the USP were observed for Pb and the lowest for Cd, Co, Cr, and Cu, both in dried plant samples and their decoctions. In the decoctions prepared from these plants were found elements such as Cd, Co, Cr, Cu, Fe, Na, Zn, and Pb. In the decoction prepared from 40 g *C. salicifolia* leaves and 40 g *C. alba* wood, the content of Cd is above the oral daily exposure value set by the USP. Hazard index (HI) for decoctions prepared from these plants exceeded the threshold (1). Given the uncertainties associated with the estimates of toxicity values and exposure factors, futures researches should address the possible toxicity in humans. Uncontrolled selling and long-term ingestion of medicinal plants can cause toxicity and interfere with the effect of drugs. Limited knowledge on the interaction potential of medicinal plants poses a challenge and public health problem in Brazil and other countries.

## 1. Introduction

Medicinal plants are widely used in the treatment of several diseases, as cosmetics, pharmaceuticals, and chemicals [1]. Medicinal plants are popular because of their low financial cost and easy access to purchase without a prescription. The use of medicinal plants and phytonutrients or nutraceuticals has increased rapidly worldwide in recent years [2]. In Brazil, the use of medicinal plants has been embraced as complementary and alternative medicines [3, 4], as well as in the UK and the rest of Europe, North America, and Australia [5]. Except for India, Bulgaria, and Nepal, few countries have medicinal plant quality and control regulations [6]. The legislation on herbal medicines was revised to prepare new Brazilian standards [7]; however, the permissible limit of heavy metal concentration in medicinal plants is not yet established.

Environmental factors that affect plant growth and development include soil, temperature, water, humidity, and pollution [8]. However, effects of some organic compounds and their products present in medicinal plants on human health are not well known. Therefore, controlling heavy metal concentrations in both medicinal plants and their decoctions or infusions should be made to ensure the safety and effectiveness of herbal products [9].

The quality of medicinal plants regarding their nutritional value and toxicity can be evaluated by the determination of macro- and microelements. Element levels of Co, Cr, Cu, Fe, Mn, Mo, Se, Zn etc. are necessary for the proper functioning of a living organism. However, excessive metal levels can be damaging to the organism [10, 11]. The determination of the element levels of As, Cd, and Pb are essential because of their toxic nature and metabolic role [12].

To date, in Brazil, medicinal plants are freely sold on the open-air markets, at fairs, and especially by herbal vendors in the streets with little or no restriction [13]. Based solely on traditional knowledge, the herb sellers or companies marketing medicinal plants do not provide information on the maximum daily dosage that can cause toxicity in children, adults, and the elderly [14]. According to a study not published by a Brazilian research group (Spectroscopy and Bioinformatics Applied to Biodiversity and Health Group, Faculty of Medicine, Brazil), the labeling of the packaging (or plastic containers) of several marketed medicinal plants has similar dosages, i.e., the preparation consists of 2 or 3 tbsp added to one liter of water (decoction process). Also, there is no maximum daily dose per weight or age.

A freely traded medicinal plant is *Cordia salicifolia* Cham. or *Cordia ecalyculata* Vell. (Boraginaceae family). In Brazilian folk medicine, *C. salicifolia* is used as an antiobesity medicine, appetite suppressant, and diuretic [13]. *C. salicifolia* is popularly known as “porangaba” or “cafezinho-do-mato” grows abundantly in the central and northeast regions of Brazil and the tropical forest areas of Argentina and Paraguay [15]. Another free-trade medicinal plant is *Chiococca alba* (L.) Hitchc.; its roots are used as diuretic, purgative, and snake bite medicine and are hydroponic [16]. *C. alba* (L.) Hitchc. (Rubiaceae), commonly known in Brazil as “Cainca” or “cipó-cruz,” is a tropical and subtropical shrub distributed in some regions of Brazil [17]. Also, *Echites peltata* is a medicinal plant marketed freely in various regions of Brazil. It is used as an anti-inflammatory and indicated for chronic ulcers, diuretic, orchitis to combat uterine cramps, and rheumatic and joint pain. It is a climbing plant with alternating leaves and large clustered flowers. Its roots have a thick and bitter bark that froths when struck with water [18], popularly called the root of Joao da Costa or Erva Santa in Brazil.

Although some medicinal plants are used as supplements, foods, and teas to assist in weight control [19], information about their mineral composition is scarce. To date, metal levels in plant species including *C. salicifolia*, *C. alba* (L.) Hitchc., and *E. peltata* and their decoction have never been assessed. This work aimed to determine the content of Cd, Co, Cr, Cu, Fe, Na, Pb, and Zn in these three different medicinal plant species *C. salicifolia*, *C. alba* (L.) Hitchc., and *E. peltata*, including their dry samples and decoctions, using the high-performance technique “inductively coupled plasma-optical emission spectrometry (ICP OES).” Metal contents detected in the dry plant samples were evaluated and compared with the permitted limits of metal impurities established by the United States Pharmacopoeia Convention (USP) (*µ*g/g) [20]. Besides, the metal content in these plant decoctions were compared with the oral permissible daily exposures established by the United States Pharmacopoeia Convention (USP) (mg/day) [20]. To the best of our knowledge, there are no scientific reports available on the human health risk assessment of some metals in these plants. Thus, Cd, Co, Cr, Cu, Fe, Na, Pb, and Zn were assessed by evaluating the chronic daily intake (CDI) and hazard quotient (HQ) to determine the hazard indices (HI) in the decoctions prepared by mixing the plant samples in water [21].

## 2. Materials and Methods

### 2.1. Samples

A total of 30 samples of various commercially available medicinal plants were randomly purchased from “Cha & Cia Produtos Naturais” in Brazil during the period of 2018-2019. The plant samples were commercially available in the plastic containers with pieces of leaves (*C. salicifolia*), wood (*C. alba* (L.) Hitchc.), and branches (*E. peltata*); each plastic container contains 100 g of the herbs raw material.

### 2.2. Sample Preparation by Microwave Digestion

#### 2.2.1. Microwave Digestion for Dry Plant Samples

For the analysis of the leaves (*C. salicifolia*), wood (*C. alba* (L.) Hitchc. (Cainca)), and branches (*E. peltata*), samples of each plant were subjected to a drying process for 6 hours in a hot oven at 45°C. Immediately after drying, accurately weighed 100 g of each sample was crushed and sieved (stainless steel sieve, 200 *μ*m granulometry). In the procedure, each sample of the plant (0.25 g) was weighed into the digestion vessels. 0.1 to 0.5 g of the sample, which is the recommended weight by the Brazilian Pharmacopoeia, was used in our experiment [22]. The digestion was performed by adding 3.0 mL of HNO_3_ (65%; Merck), 1.0 mL of high-purity water (18 MΩ·cm; Milli-Q, Millipore, Bedford, MA, USA), and 2.0 mL of H_2_O_2_ (35%; Merck) to the sample. All vessels with the samples of plants were placed in the microwave digestion system (Speedwave 4-Microwave Digestion System; BERGHOF Products + Instruments GmbH), according to the digestion program instruction depicted in Table 1 [23, 24]. The microwave oven heating program was carried out in three steps.

#### 2.2.2. Microwave Digestion for Plant Teas

Recent methodology of microwave digestion for plant teas is given in Ref. [23, 24]. As an experimental quality control to the methodologies, each tablespoon corresponds to 10 g of the powder sample. In the labeling of the plastic packaging of medicinal plants sold by companies, the decoction process happens by adding 2 or 3 tablespoons in a liter of water. However, in our experiment, we consider 1, 2, and 4 tablespoons in a liter of water. We filled three glass beakers (total capacity 2000 mL) with 1000 mL of high-purity water, and then 10 g, 20 g, and 40 g of each plant sample were added, respectively. The samples were covered and boiled by using a heating plate for 10 min. Next, the samples were covered and left at room temperature of 25 min.

After cooling, the extract was filtered through a quantitative filter paper. After this, in each of the tea samples was added aliquots of 1 mL of concentrated HNO_3_ (65%; Merck) and 0.5 mL of H_2_O_2_ (35%; Merck). All analyses were carried out in triplicate, and analytical blanks were also prepared following the same procedure used for the samples. Subsequently, the tea samples were placed in the digestion vessels of microwave digestion system (Speedwave 4-Microwave Digestion System; BERGHOF Products + Instruments GmbH), according to the operating program specified in Table 2. The final volume was adjusted to 10 mL with ultrapure water (Milli-Q Biocel Water Purification System; Millipore, Germany).

### 2.3. Calibration Procedure

The calibration curves and stock solution to mineral detection were set up with high-purity water and nitric acid. The concentrations of the different elements in these samples were measured using the corresponding multielement standard solution (100 mg/L; Specsol, São Paulo, Brazil) containing Co, Cu, Fe, Na, and Zn and monoelement standard solution (100 mg/L, Specsol, São Paulo, Brazil) containing Ca, Cr, and Pb. A nine-point calibration curve was generated using the following concentrations: 0.01, 0.025, 0.05, 0.1, 0.25, 0.5, 1.0, 2.0, and 4.0 ppm of the element standards.

### 2.4. Elemental Analysis by ICP OES Technique

After microwave-assisted digestion with a mixture of nitric acid and hydrogen peroxide, a clear solution was obtained and the analytes were determined by ICP OES (iCAP 6300 Duo; Thermo Fisher Scientific, Bremen, Germany).  Table 3 shows the instrumental conditions maintained for the analysis of Cd, Co, Cr, Cu, Fe, Na, Pb, and Zn. Parameters of the analytical curve for all studied elements are presented in Table 4.

Regarding validation, the detection limit (LOD) was calculated as three times the standard deviation of the blank expressed in concentration divided by slope of the analytical curve, and the limit of quantification (LOQ) was obtained ten times the standard deviation of the blank divided by slope of the analytical curve according to IUPAC [25]. The external calibration equation and their respective correlation coefficients (*R*^*2*^) are presented in Table 4. The values of LOD were in the range of 0.2–2.3 (*μ*g/L), and the values of LOQ were 0.7–7.7 (*μ*g/L) as shown in Table 4.

### 2.5. Validation of Methods

The accuracy of the measurements was determined by a spike recovery test by adding 0.5 mg/kg and 1.0 mg/kg of each metal in dry plant samples. The results were obtained as the average of three replicates of each sample and are shown in Table 5. As can be seen, the method has good accuracy and the recoveries were between 91% and 127%.

### 2.6. Comparative Study

The commercialization of medicinal plants in Brazil occurs in the form of tablets, capsules, powders, teas, extracts, and fresh or dried plants. Thus, the metal contents quantified in the dry plant samples (*µ*g/g) were compared with oral concentration obtained from the permitted concentration of elemental impurities in drug substances and excipients established by the United States Pharmacopoeia Convention (USP) [20]. Also, the metal concentration detected in the decoction processes of the plants (*µ*g/L) was evaluated and compared with the oral permitted daily exposures (EDPs) by the United States Pharmacopoeia Convention [20].

Acceptable levels of impurities for components (drug substances and excipients) in drug products are established by the United States Pharmacopoeia Convention (USP). USP considers oral permitted daily exposures (EDPs) based on the amount of elemental impurities in medicinal products. Elemental impurities include catalysts and environmental contaminants that may be present in drug substances, excipients, or drug products [20].

### 2.7. Estimation of Human Health Risk Assessment

Based on the above information, a risk-based control strategy is appropriate to ensure compliance with this standard. Thus for the risk assessment, the presence of elements such as cadmium (Cd), cobalt (Co), chromium (Cr), copper (Cu), iron (Fe), sodium (Na), lead (Pb), and zinc (Zn) in substances, excipients, or medications is considered.

In order to estimate human health risk associated with heavy metals in teas prepared from each medicinal plant in different concentrations (one tablespoons = 10 g of each sample, two tablespoons = 20 grams of each sample, and four tablespoons = 40 grams of each sample), chronic daily intake (CDI) was calculated first using the following equation [21]:

(1) $$\begin{array}{c} \text{CDI}=\frac{\text{CP}\times \text{IR}\times \text{EF}\times \text{ED}}{\text{Bw}\times \text{AT}}, \end{array}$$

where CDI is the chronic daily intake by ingestion (mg/kg/day) to which consumers might be exposed [21]. CP is the chemical concentration in a plant (mg/L); in this study, values obtained by ICP OES. IR is the ingestion rate (L/day) [26]; in this study, 1 L/day for adults. EF is the exposure frequency (days/year); in this study, 90 days/year. ED is the exposure duration (years); in this study, 30 years for adults. Bw is the body weight (kg); in this study, 70 kg for adults [27]. The AT is the averaging time for noncarcinogens (period over which the exposure is averaged (days)); in this study, AT = ED × 90 days = 2700 days [21].

Determination of accurate data from chronic daily intake (CDI) is complex, and it usually requires exposure frequency (180–350 days) and duration among individuals (30, 68.35, and 70 years), body weight (60–70 kg), ingestion rate of vegetables or liquids (1-2 L/day), and average time (exposure days within whole lifetime) [21].

#### 2.7.1. Hazard Quotient (HQ)

The risk of consumption of metal-contaminated medicinal plants in human health was characterized by hazard quotient (HQ), as shown in equation (2). HQ is the ratio of determined dose of a pollutant to the reference dose (RfD). If HQ > 1, the population will experience health risk, while HQ < 1 represents the acceptable level (population will pose no risk). An estimate of the potential hazard to human health (HQ) through consumption of medicinal plants grown in metal-contaminated soil is calculated in the following equation:

(2) $$\begin{array}{c} \text{HQ}=\frac{\text{CDI}}{\text{RfD}}, \end{array}$$

where CDI is the chronic daily intake (mg/kg/day) in each plant and different concentrations (equation (1)) and RfD is the chronic oral reference dose for the metal(loid)s (mg/kg of body weight per day) [28], which does not cause a lifetime deleterious effect.

#### 2.7.2. Hazard Index (HI)

The hazard index is the sum of the hazard quotients; it is calculated due to the presence of more than one heavy metal in the sample (equation (3)). USEPA developed the hazard index (HI) in 1989 [29]. That is, the hazard index assumes that the magnitude of the adverse effect will be proportional to the sum of several element exposures. In many studies, risk is assessed by assuming the sum of all elements quantified from a specific sample, in which chemical(s) concentration(s) have been measured by equipment such as inductively coupled plasma mass spectrometry (ICP MS) [30, 31] or inductively coupled plasma-optical emission spectrometry (ICP OES) [32].

(3) $$\begin{array}{c} \text{HI}=\sum \left(\text{HQ}\right)={\text{HQ}}_{\text{Cd}}+{\text{HQ}}_{\text{Co}}+{\text{HQ}}_{\text{Cr}}+{\text{HQ}}_{\text{Cu}}+{\text{HQ}}_{\text{Fe}}+{\text{HQ}}_{\text{Na}}+{\text{HQ}}_{\text{Pb}}+{\text{HQ}}_{\text{Zn}}. \end{array}$$

If ∑(HQ) < 1, it is assumed that chronic risk is unlikely to occur due to tea ingestion of this plant.

### 2.8. Statistical Analysis

Results obtained were reported using the Origin 9.0 software (OriginLab Corporation, Northampton, MA, USA.). Concentrations were expressed as mean ± standard deviation, minimum, and maximum values. One-way analysis of variance (ANOVA) was used to test for differences in element levels in the dry plant samples. The association between elements was examined using Person's correlation coefficient. Significant correlations were declared weak (*r* < 0.3), moderate (*r* from 0.3 to 0.7), or strong (*r* > 0.7). Results were considered significant if *p* ≤ 0.05.

## 3. Results and Discussion

### 3.1. Dry Plant samples

As can be seen in Table 6, the concentration of the metals in the leaves of *C. salicifolia* in *µ*g/g decreases in the order of Na > Fe > Zn > Cu > Pb > Cr > Cr, whereas in the wood of *C. alba* was in the order of Fe > Zn > Na > Cu > Pb > Cr > Cd, and in the branches of *E. peltata* was Fe > Na > Cu > Zn > Pb > Cr > Co.

According to the ANOVA statistical analysis, the highest concentration of metals is found in the leaves of *C. salicifolia* (66.36 ± 2.9), followed by the wood of *C. alba* (37.83 ± 1.21), and lastly in the branches of *E. peltata* (33.05 ± 1.10). There are differences between the concentrations of the metals found in plants. On the other hand, Table 7 presents Pearson's correlation coefficients for Cd, Co, Cr, Cu, Fe, Na, Pb, and Zn in the samples of medicinal plants. The matrix shows the strength of the linear relationships between each pair of variables. In this study, a significant moderate and positive correlation was determined between Zn and Cd (*r* = 0.688), Pb and Cu (*r* = 0.58871), Pb and Fe (*r* = 0.4772), Zn and Na (*r* = 0.3160), and Cu and Cd (*r* = 0.3054). Furthermore, the correlation analysis showed a significant strong and positive correlation between Na and Cd (*r* = 0.9055), Fe and Co *(r* = 0.8905), Cu and Cr (*r* = 0.8049), Pb and Cr (*r* = 0.9535), and Zn and Cu (*r* = 0.9007) in the samples. There was a significant moderate and negative correlation between Pb and Cd (*r* = −0.5899), Cr and Cd (*r* = −0.3192), Na and Co (*r* = −0.5026), Na and Cr (*r* = −0.6912), Fe and Cu (*r* = −0.4295), and Cu and Co (*r* = −0.7933). In addition, there was a significant strong and negative correlation between Co and Cd (*r* = −0.8220), Cr and Cd (*r* = −0.3192), Fe and Cd (*r* = −0.9911), Zn and Co (*r* = −0.9791), Na and Fe (*r* = −0.8409), and Pb and Na (*r* = −0.8769).

The content of Cd ranged between 0.7 ± 0.1 *µ*g/g in *E. peltata* branches and 0.3 ± 0.06 *µ*g/g in *C. alba* wood (Table 6). The content of Cd in *E. peltata* branches is below the limit of detection (Table 6). The permitted concentration of impurities such as Cd (oral concentration) set by the USP was 5.0 *µ*g/g [33], and after comparison with other medicinal plants, it is found that the Cd content in the plants used in this study is below this limit [33].

The content of Co in *C. salicifolia* leaves and *C. alba* wood is below the limit of detection (Table 6). On the other hand, the amount of Co in the branches of *E. peltata* analyzed was 0.4 ± 0.01 *µ*g/g. The permitted concentration of impurities such as Co established by the USP in drug substances and excipients was 5 *µ*g/g. Thus, Co concentration in the branches of *E. peltata,* obtained in the present study, was below the value established by USP [20].

The lowest content of Cr was in *C. salicifolia* leaves, that is, 0.9 ± 0.1 *µ*g/g, and the maximum concentration of 1.3 ± 0.1 *µ*g/g was in *C. alba* wood and that of 1.0 ± 0.03 *µ*g/g was in branches of *E. peltata* (Table 6). Thus, the Cr content in these plant samples is below the permitted concentration of impurities for drug substances and excipients established by the USP (1100 *µ*g/g) [20].

As shown in Table 6, the range of Cu content varied between 9.5 ± 0.8 *µ*g/g in *C. salicifolia* leaves, 13 ± 1.1 *µ*g/g in *C. alba* wood, and 7.3 ± 1.0 *µ*g/g in branches of *E. peltata*. The Cu concentration in these plant samples was below the permitted concentration of impurities for drug substances and excipients set by the USP (300 *µ*g/g).

The concentration of Fe in the leaves of *C. salicifolia*, the wood of *C. alba,* and the branches of *E. peltata* were 135 ± 10 *µ*g/g, 176 ± 1.7 *µ*g/g, and 225 ± 8.3 *µ*g/g, respectively (Table 6). There is no concentration limit of elemental impurities such as Fe in drug substances and excipients established by USP [20].

According to Table 6, the leaves of *C. salicifolia* has the highest Na content, that is, 135 ± 10 *µ*g/g, *C. alba* wood 176 ± 1.7 *µ*g/g and *E. peltata* branches 225 ± 8.3 *µ*g/g. However, the Na concentration limit in drug substances and excipients has not yet been set by the USP [20].

Table 6 shows that the concentration of Pb in the leaves of *C. salicifolia* was 1.8 ± 0.2 *µ*g/g, 1.4 ± 0.005 *µ*g/g for *C. alb*a wood, and 3.6 ± 0.02 *µ*g/g for branches of *E. peltata*. The permitted concentration of Pb in drug substances and excipients established by the USP was 0.5 *µ*g/g [20]. Thus, the Pb concentration in all these plants was above the permissible limit.

The concentration of Zinc element was much higher in the wood of *C. alba,* that is, 85 ± 5.9 *µ*g/g, than in the leaves of *C. salicifolia* 68 ± 8.0 *µ*g/g, followed by 5.7 ± 0.2 *µ*g/g in the branches of *E. peltata* (Table 6). The concentration limit of Zn in drug substances and excipients is not yet established by the USP [20].

### 3.2. Plant Teas

Table 8 shows the results of teas prepared by 10 min decoction obtained by mixing 10 g, 20 g, and 40 g of the dried plant samples in glass beakers containing 1000 mL of water. The results obtained from the decoction process performed for the three plants and the respective amounts of tablespoons used to make the teas in *µ*g/L are presented in Table 8. In addition, the quantified metals in the plants were compared with the oral permitted daily exposure (EDP) values established by the USP (mg/day) [20]. In comparison (Table 8), the levels of Cd and Co in some teas were low (below the detection limit).

In the decoction process performed by adding two tablespoons (10 g of each plant sample) in one liter of water (Table 8), the concentration of Cd in *C. salicifolia* leaves was 2.0 ± 0.1 *µ*g/L, which was well below the value of permissible daily oral exposure (PDEs) established by the USP for Cd (5 *µ*g/day). For four tablespoons (40 g), the Cd concentration found in the tea of *C. salicifolia* leaves was 15 ± 0.1 *µ*g/L, and for the tea of *C. alba* wood, it was 7.6 ± 0.1 *µ*g/L. After comparing the metal concentration in the teas of the plants (40 g) with those proposed by the USP, it is found that *C. salicifolia* and *C. alba* had Cd above the permissible oral daily exposure value [20].

The concentration of Co in the decoction process performed by adding four tablespoons (40 g) of branches of *E. peltata* was 2.9 ± 0.2 *µ*g/L (Table 8). The daily oral intake limit set by the USP for Co was 50 mg/day, and the Co concentration in the *E. peltata* branches was below this limit [20].

In this study, Cr concentration from the plants in the decoction process varied between 0.7 ± 0.4 and 23 ± 0.3 *µ*g/L (Table 8), and the permissible oral daily exposure (PDE) value set by USP is 11,000 *µ*g/day [17]. On the other hand, the concentration of Cu from the plants varies widely between 33 ± 0.4 and 374 ± 0.3 *µ*g/L, and the recommended PDE by the USP is 3000 *µ*g/day. In *C. salicifolia* leaves, *C. alba* wood, and *E. peltata* branches studied, the concentration of Cr and Cu is much lower than the permissible levels.

As shown in Table 8, the concentration of Fe in teas ranges between 668 ± 8.6 *µ*g/L and 4876 ± 13 *µ*g/L in all parts of the plants. There is no permissible oral daily exposure limit for Fe set by the USP. However, the maximum allowed concentration of Fe in drinking water is 1000 *µ*g/L, according to the WHO report [34]. After comparing the concentration of Fe in the plants with those proposed by the WHO, we found that only *E. peltata* branches (1 tablespoon = 10 g) had Fe content below this limit, while all other plants had Fe content above this limit. To date, there is no iron poisoning found in humans due to the intake of iron-rich plants.

However, iron poisoning can be caused by taking high doses of iron supplements (pediatric patients) for prolonged periods or by taking a single overdose [35].

The concentration of Na in teas of plant samples varied from 668 ± 6.2 to 7404 ± 43 *µ*g/L (Table 8). The permissible oral daily exposure (PDEs) by the USP for Na has not yet been established. There is no agreement on the minimum daily requirement, and these recommendations have been controversial. However, a high level of sodium in drinking water seriously aggravates chronic congestive heart failure [36]. The concentration of Na is highest in human's and cow's milk, that is, 0.180 and 0.770 *µ*g/L [37], respectively. The WHO suggests consuming 2 *µ*g of sodium per day [38]. After comparing the metal limit in the studied medicinal plants with those proposed by the WHO, it is found that all parts of the plants had Na above this limit.

The level of Pb in teas of plants varied from 9.2 ± 1.1 to 151 ± 0.5 *µ*g/L (Table 8). The permissible value (PDE) proposed by USP is 5 *µ*g/day, and the concentration values were all above the recommended value [20]. Lead is classified as a carcinogen by the Agency for Toxic Substances and Disease Registry (ATSDR) [39]. Besides, according to the recommendation of the Joint FAO/WHO Expert Committee on Food Additives, the weekly intake of lead should not exceed 25 *µ*g/kg of body weight per week for humans [40, 41]. Based on such cited evidence [40, 41], prolonged ingestion or large daily doses of tea from these plant types can cause toxicity due to the high accumulation of Pb.

The concentration of Zn in the teas of these plants ranged from 36 ± 0.3 to 2405 ± 4.3 *µ*g/L (Table 8). The permissible oral daily exposure of Zn by USP has not yet been established. The maximum permissible limit of zinc in water reported by the WHO is 5000 *µ*g/L [42]. In our study, the concentration of zinc is lower than the maximum permissible limit. On the other hand, according to the Agency for Toxic Substances and Disease Registry (ATSDR), the levels of zinc that produce adverse health effects are much higher than the recommended dietary allowance (RDAs) for zinc of 8–11 mg/day for adults. Thus, the ingestion of large amounts of zinc into the body through food and water can also affect health [43].

### 3.3. Discussion on Human Health Risk Assessment

The chronic daily intake (CDI) and oral reference dose (RfD) of each heavy metal in plant teas are summarized in Table 9. The CDI values of 0.0000285 mg/kg/day and 0.000214 mg/kg/day for adults, respectively, were recorded for Cd in *C. salicifolia* leaves, while 0.000108 mg/kg/day was recorded for adults in *C. alba* wood. Also, the cadmium CDI values for adults were below the oral reference dose for Cd (0.0005 mg/kg/day). It is assumed that the consumption of the plant teas is 1 L per day. However, the consumption of more significant quantities of tea, four or more cups compared with 1 or less, may be detrimental to health. According to studies, cadmium induces cancers in animals and humans [44]. Cadmium toxicity affects photochemical and nutrient element composition of lettuce (*Lactuca sativa* L.) [45].

In Table 9, the CDI value of cobalt for adults (0.00004142 mg/kg/day) was below oral reference dose (0.01 mg/kg/day). Toxicity caused by the ingestion of cobalt through food and water is rare. However, excessive exposure is related to the induction of various adverse health effects [46].

The CDI values of Cr for adults ranged within 0.00001–0.00021 mg/kg/day in teas of *C*. salicifolia leaves, 0.0000414–0.000328 mg/kg/day in teas of *C. alba* wood, and 0.000051–0.000134 mg/kg/day in teas of *E. peltata* branches. All CDI values are below oral reference dose (0.0003 mg/kg/day), except for the teas of *C. alba* wood. Cr is an essential nutrient for humans, and foodstuffs (ingestion) generally contain extremely low chromium levels. Chromium (III) can be toxic if consumed in large doses [47].

The CDI values of Cu for adults in teas of *C. salicifolia* leaves (0.00107–0.00420 mg/kg/day), teas of *C. alba* wood (0.00135–0.005342 mg/kg/Day), and teas of *E. peltata* branches (0.000471–0.002571 mg/kg/day) are below the oral reference dose (0.01 mg/kg/day). Copper plays a critical role in the biochemistry of all living organisms. However, Cu ingested in large amounts in the diet may cause toxicity [48].

The CDI values of Fe for adults ranged within 0.0196–0.0693 mg/kg/day in teas of *C*. *salicifolia* leaves, 0.02131–0.0696 mg/kg/day in teas of *C. alba* wood, and 0.00954–0.0360 mg/kg/day in teas of *E*. peltata branches. All CDI values of Fe are above the oral reference dose (0.007 mg/kg/day). Iron is an essential trace element in living organisms. This element occurs as a natural constituent in plants. However, iron toxicity from intentional (suicide attempts) or accidental ingestion (potent adult preparations) is a common poisoning [49].

In Table 9, the CDI values of Na for adults varied in the range between 0.0789 and 0.24860 mg/kg/day in teas of *C. salicifolia* leaves, 0.0249 and 0.0351 mg/kg/day in teas of *C. alba* wood, and 0.0111 and 0.0351 mg/kg/day in teas of *E. peltata* branches. All CDI values of Na for adults are above the values of oral reference dose (0.05 mg/kg/day). Salt poisoning is an intoxication resulting from the excessive intake of sodium through diet. Fatal ingested sodium doses were estimated to be less than 25 g [50].

The CDI values of Pb for adults in teas of *C*. *salicifolia* leaves (0.000185–0.00107 mg/kg/day) and teas of *C. alba* wood (0.000471–0.002157 mg/kg/day) were all below oral reference dose (0.0035 mg/kg/day), while that of in the tea of *E*. *peltata* branches (0.000131–0.1265 mg/kg/day) was above RfD values. Exposure to lead occurs via inhalation and ingestion of lead-contaminated food and water [51]. A high concentration of Pb can affect the central nervous, cardiovascular, and especially the immune systems [43]. Even the minimal presence of lead in the human body causes various health damages [52].

The CDI values of Zn for adults in the teas of *C. salicifolia* leaves, *C. alba* wood, and *E. peltata* branches varied from 0.00824 to 0.0332 mg/kg/day, 0.00828 to 0.0343 mg/kg/day, and 0.00051–0.001771 mg/kg/day, respectively. These CDI values of zinc for adults in the plant teas were below the oral reference dose (0.3 mg/kg/day). Zinc is one crucial trace element in biological systems. The principal sources of exposure to zinc include the ingestion of food and drinking water [53]. Zinc poisoning can occur from dietary supplements by accident; ingestion of zinc greater than 150 mg per day is toxic [54].

Table 10 contains the results of the analysis on hazard quotient (HQ) and total hazard index (HI) of the heavy metals for the human population (mg/kg/day). The HQ of Cd for adults was 0.05714 and 0.4285 in the teas of *C. salicifolia* leaves and 0.21714 in the teas of *C. alba* wood. The HQ values of Cd for adults in the teas of these plants were below 1.

The HQ for Co for adults is 0.00414 in *E. peltata* branch tea, which is less than 1 (Table 10). The HQ for Cr for adults varies from 0.0333 to 1.095 in the tea of the plants (Table 10). Only the HQ of Cr in *C. alba* wood was higher than 1. The HQ value of Cu for adults in the teas of the plants varied widely between 0.0471 and 0.5342 (Table 10). All HQ values of Cr in the teas of the plants were over 1.

As shown in Table 10, the HQ value of Fe for adults in the teas of the plants range from 1.3632 to 9.951, which is higher than 1.

As shown in Table 10, the HQ values of Na for adults in the teas of the plant samples varied from 0.1908 to 4.9725. The HQ value of Na for adults in the teas of *C. salicifolia* leaves was 1.5785 (one tablespoon), 2.6948 for *C. alba* wood (two tablespoons), and 4.9725 for *E. peltata* branches (four tablespoons); all these values are above 1.

The HQ values of Pb for adults in the teas of the plant samples were in the range of 0.0375–0.6163 (Table 10). All values are greater than 1.

The HQ o Zn for adults varied from 0.0017 to 0.1145 in the teas of the plants (Table 10), and thus the HQ values of Zn for adults were below the oral reference dose (0.3 mg/kg/day).

In Table 10, the total hazard index (HI) of heavy metals is high for *C. salicifolia* leaves, followed by *C. alba* wood and branches of *E. peltata*. All the total hazard index (HI) values recorded in this study were above 1.

Thus, the long-term health risk is high and the noncarcinogenic adverse effect is not negligible. The toxicity from medicinal plants depends on several factors, including the dose, route of exposure, and chemical species, as well as the age, gender, genetics, and nutritional status of the exposed individuals.

## 4. Conclusions

The concentrations of Cd, Co, Cr, and Cu in the dry *C. salicifolia* leaves, *C. alba* wood, and *E. peltata* branches were below the oral concentration of elemental impurities established by the United States Pharmacopeia Convention (USP).

There is no concentration limit of elemental impurities such as Fe, Na, and Zn in drug substances and excipients established by the USP. All parts of plants had Pb at a concentration above the oral concentration of elemental impurities established by the United States Pharmacopoeia Convention (USP). Thus, taking the plant in capsules or tablets can be harmful to health.

The teas of 40 g *C. salicifolia* leaves and *C. alba* wood had Cd above the oral daily exposure value set by the USP. Other plants had Co, Cr, and Cu below the oral permitted daily exposure (EDP) values established by the USP (mg/day). The permissible oral daily exposures (PDEs) for Fe, Na, and Zn have not yet been set by the USP. The level of Pb in teas of plants was above the recommended value by the USP.

Concerning human health risk assessment calculus, the CDI values of Cd, Co, Cr, Cu, and Zn for adults in plant teas were all below the oral reference dose. On the other hand, the CDI values of Cr (tea with four tablespoons of *C. alba*), Fe, Na, and Pb for adults were above the oral reference dose, which resulted in a hazard index above 1. These data show that the ingestion of these plants in decoction form could be toxic for adults. Although there are uncertainties associated with the estimates of toxicity values such as the averaging time for noncarcinogens (AT) and exposure frequency (EF), long-term health risk and the noncarcinogenic adverse effect are not negligible.

Medicinal plants are not classified as drugs by the Brazil law. The ingestion of medicinal plants and their products requires strict control of the presence of heavy metals, the dosage labeling, contraindications, manufacturing techniques, and principally a list of all composition. Although medicinal plants are effective in treating some diseases, their continued and uncontrolled use is of concern in many countries, including Brazil.

## Figures and Tables

**Table 1:** Heating program for microwave digestion of dry plant samples.

Step	Temperature (°C)	Pressure (bar)	*T*_Ramp_ (min)	*T*_Hold_ (min)	Power (W)
1	160	35	1	5	1120
2	190	35	1	10	1120
3	50	0	1	10	0

**Table 2:** Heating program for microwave digestion of by tea.

Step	Temperature (°C)	Pressure (bar)	*T*_Ramp_ (min)	*T*_Hold_ (min)	Power (W)
1	100	30	1	5	840
2	150	30	1	10	1120
3	50	25	1	1	0

**Table 3:** ICP OES instrumental parameters.

Parameters	ICP OES
Radiofrequency power	1150 W
Pump flow	50 rpm
Plasma argon flow	12.00 L min^−1^
Auxiliary argon flow	0.50 L min^−1^
Nebulizer gas flow	0.70 L min^−1^
Plasma view	Axial
Analytes	Wavelength (nm)
Cd	228.8
Co	228.6
Cr	283.5
Cu	324.7
Fe	259.9
Na	588.9
Pb	220.3
Zn	213.8

**Table 4:** Calibration equations (equations (*A* = aC + I)^*∗*^, correlation coefficients, LOD, and LOQ obtained by external calibration).

Analyte	External calibration equation	*R* ^2^	LOD (*μ*g/L)	LOQ (*μ*g/L)
Cd	*A* = 18394 C + 140	0.9999	0.2	0.7
Co	*A* = 6790 C + 70	0.9999	0.5	1.7
Cr	*A* = 18636 C + 92	0.9999	0.3	1.1
Cu	*A* = 31716 C + 273	0.9999	0.4	1.3
Fe	*A* = 11560 C + 149	0.9997	0.8	2.5
Na	*A* = 377454 C − 4767	0.9997	0.9	3
Pb	*A* = 947 C + 11	0.9998	2.2	7.4
Zn	*A* = 11563 C + 183	0.9996	1.2	4.2

^*∗*^ *A* = absorbance; a = slop; C = concentration (*μ*g·L^−1^); *I* = intercept.

**Table 5:** Validation of methods using the spike concentration of plant samples.

Analyte	Spike concentration (%)
Cd	95–100
Co	94–98
Cr	100–04
Cu	98–106
Fe	95–105
Na	108–127
Pb	91–95
Zn	97–103

**Table 6:** Comparison of quantified metals in the medicinal plants with the permitted limits of metal impurities established by the USP (*µ*g/g).

Metals	Dry plant samples (*µ*g/g ± SD)			Oral concentration (*µ*g/g) USP
	*Cordia salicifolia* leaves	*Chiococca alba* (*L.*) *Hitchc*. wood	*Echites peltata* branches	
Cd	0.7 ± 0.1	0.3 ± 0.06	<LOD	5^*∗*^
Co	<LOD	<LOD	0.4 ± 0.01	5
Cr	0.9 ± 0.1	1.3 ± 0.1	1.0 ± 0.03	1100
Cu	9.5 ± 0.8	13 ± 1.1	7.3 ± 1.0	300
Fe	135 ± 10	176 ± 1.7	225 ± 8.3	—
Na	315 ± 16	23 ± 2.8	22 ± 1.3	—
Pb	1.8 ± 0.2	4.1 ± 0.1	3.0 ± 0.02	0.5
Zn	68 ± 8.0	85 ± 5.9	5.7 ± 0.2	—

<LOD, analyte concentration was below the limit of detection. ^*∗*^New values for Cadmium established in Ref. [33].

**Table 7:** Pearson's correlation coefficients for Cd, Co, Cr, Cu, Fe, Na, Pb, and Zn in the samples of the medicinal plants.

	Cd	Co	Cr	Cu	Fe	Na	Pb	Zn
Cd	1							
Co	−0.8220	1						
Cr	−0.3192	−0.2774	1					
Cu	0.3054	−0.7933	0.8049	1				
Fe	−0.9911	0.8905	0.1901	−0.4295	1			
Na	0.9055	−0.5026	−0.6912	−0.1276	−0.8409	1		
Pb	−0.5899	0.0251	0.9535	0.5887	0.4772	−0.8769	1	
Zn	0.6888	−0.9791	0.4671	0.9007	−0.7792	0.3160	0.1790	1

**Table 8:** Metal concentration in the plant samples compared with the oral permissible daily exposures established by the USP (mg/day).

Analyte	Tea with one tablespoon (10 g) (*µ*g/L ± SD)			Tea with two tablespoons (20 g) (*µ*g/L ± SD)			Tea with four tablespoons (40 g) (*µ*g/L ± SD)			Oral PDE (*µ*g/day), USP
	Plant 1	Plant 2	Plant 3	Plant 1	Plant 2	Plant 3	Plant 1	Plant 2	Plant 3	
Cd	<LOD	<LOD	<LOD	2.0 ± 0.1	<LOD	<LOD	15 ± 0.1	7.6 ± 0.1	<LOD	5
Co	<LOD	<LOD	<LOD	<LOD	<LOD	<LOD	<LOD	<LOD	2.9 ± 0.2	50
Cr	0.7 ± 0.4	2.9 ± 0.7	<LOD	4.6 ± 0.2	9.8 ± 0.7	3.6 ± 0.9	14 ± 0.3	23 ± 0.3	9.4 ± 0.3	11000
Cu	75 ± 0.5	95 ± 1.2	33 ± 0.4	143 ± 0.04	176 ± 1.0	81 ± 0.2	294 ± 0.3	374 ± 0.3	180 ± 0.8	3000
Fe	1376 ± 10	1492 ± 30	668 ± 8.6	2216 ± 8.4	2473 ± 4.1	1379 ± 4.2	4851 ± 11	4876 ± 13	2524 ± 11	—
Na	5525 ± 53	1745 ± 30	781 ± 8.2	9432 ± 28	2461 ± 45	668 ± 6.2	17404 ± 43	2385 ± 21	1151 ± 4.6	—
Pb	13 ± 0.7	33 ± 1.5	9.2 ± 1.1	33 ± 0.3	83 ± 0.7	31 ± 1.2	75 ± 1.3	151 ± 0.5	68 ± 1.2	5
Zn	577 ± 5.3	580 ± 6.9	<LOD	1065 ± 1.4	1114 ± 2.5	36 ± 0.3	2324 ± 6.8	2405 ± 4.3	124 ± 11	—

<LOD, analyte concentrations were below the limits of detection; SD, standard deviation. Plant 1: *Cordia salicifolia* leaves; Plant 2: *Chiococca alba* wood; Plant 3: *Echites peltata* branches.

**Table 9:** The chronic daily intake of heavy metals (CDI).

Analyte	Tea with one tablespoon (10 g)			Tea with two tablespoons (20 g)			Tea with four tablespoons (40 g)			Oral reference dose (RfD) for heavy metals (mg/kg/day)
	Plant 1 (CDI)	Plant 2 (CDI)	Plant 3 (CDI)	Plant 1 (CDI)	Plant 2 (CDI)	Plant 3 (CDI)	Plant 1 (CDI)	Plant 2 (CDI)	Plant 3 (CDI)	
Cd	0	0	0	0.0000285	0	0	0.000214	0.000108	0	0.0005^*∗*^
Co	0	0	0	0	0	0	0	0	0.0000414	0.01^*∗*^
Cr	0.00001	0.0000414	0	0.0000657	0.00014	0.000051	0.00021	0.000328	0.000134	0.0003^*∗*^
Cu	0.00107	0.00135	0.000471	0.002042	0.002514	0.001157	0.00420	0.005342	0.002571	0.01^*∗*^
Fe	0.0196	0.02131	0.00954	0.0316	0.0353	0.0197	0.0693	0.0696	0.0360	0.007^*∗∗*^
Na	0.0789	0.0249	0.0111	0.1347	0.0351	0.0351	0.24860	0.0340	0.0164	0.05^*∗*^
Pb	0.000185	0.000471	0.000131	0.000471	0.00118	0.1265	0.00107	0.002157	0.000971	0.0035^*∗*^
Zn	0.00824	0.00828	0	0.0154	0.0154	0.00051	0.0332	0.0343	0.001771	0.3^*∗*^

^*∗*^Source: ATSDR: Oral reference dose *RfD* for heavy metals (2019) [28]; ^*∗∗*^source: USEPA IRIS (US Environmental Protection Agency) (2011) [21]. Plant 1: *Cordia salicifolia* leaves; Plant 2: *Chiococca alba* wood; Plant 3: *Echites peltata* branches.

**Table 10:** Hazard quotients (HQ) and total hazard index (HI) of the heavy metals.

Analyte	Tea with one tablespoon (10 g)			Tea with two tablespoons (20 g)			Tea with four tablespoons (40 g)		
	Plant 1 (HQ)	Plant 2 (HQ)	Plant 3 (HQ)	Plant 1 (HQ)	Plant 2 (HQ)	Plant 3 (HQ)	Plant 1 (HQ)	Plant 2 (HQ)	Plant 3 (HQ)
Cd	0	0	0	0.05714	0	0	0.4285	0.21714	0
Co	0	0	0	0	0	0	0	0	0.00414
Cr	0.0333	0.1380	0	0.2190	0.4666	0.1740	0.6666	1.095	0.4476
Cu	0.1071	0.1357	0.0471	0.2042	0.2514	0.1157	0.420	0.5342	0.2571
Fe	2.8081	3.0448	1.3632	4.5224	5.0469	2.8142	9.90	9.9510	5.1510
Na	1.5785	0.4985	0.2231	2.6948	0.7031	0.1908	4.9725	0.6814	0.3288
Pb	0.0530	0.1346	0.0375	0.1346	0.3387	0.1265	0.3061	0.6163	0.2775
Zn	0.0027	0.0276	0	0.0507	0.0530	0.0017	0.1106	0.1145	0.0059
HI	4.5827	3.9792	1.6709	7.8257	6.8597	3.4229	16.432	13.420	6.420

Plant 1: *Cordia salicifolia* leaves; Plant 2: *Chiococca alba* wood; Plant 3: *Echites peltata* branches.

## Data Availability

The data used to support the findings of this study are available from the corresponding author upon request.
